# Ultrasonography is an effective tool for the evaluation of traumatic vertebral artery injuries distal to fourth cervical vertebra in the emergency room

**DOI:** 10.1186/s12891-023-06426-6

**Published:** 2023-04-22

**Authors:** Yuyu Ishimoto, Hiroshi Iwasaki, Mayumi Sonekatsu, Shizumasa Murata, Takuhei Kozaki, Hiroshi Hashizume, Shunji Tsutsui, Masanari Takami, Keiji Nagata, Kazuhiro Hira, Seiya Kato, Hiroshi Yamada

**Affiliations:** 1 Department of Orthopedic Surgery, Wakayama Medical University, 811-1 Kimiidera, Wakayama City, Wakayama 641-8510 Japan; 2 Department of Emergency and Critical Care Medicine, Wakayama Medical University, 811-1 Kimiidera, Wakayama City, Wakayama 641-8510 Japan

**Keywords:** Ultrasonography, Vertebral artery, Cervical spine injury

## Abstract

**Background:**

This study aimed to determine the feasibility of ultrasonography in the assessment of cervical vertebral artery (VA) injury as an alternative to computed tomography angiography (CTA) in the emergency room.

**Methods:**

We analyzed 50 VAs from 25 consecutive patients with cervical spine injury that had been admitted to our emergency room. Ultrasonography and CTA were performed to assess the VA in patients with cervical spine injury. We examined the sensitivity and specificity of ultrasonography compared with CTA.

**Results:**

Among these VAs, six were occluded on CTA. The agreement between ultrasonography and CTA was 98% (49/50) with 0.92 Cohen's Kappa index. The sensitivity, specificity, and positive and negative predictive values of ultrasonography were 100%, 97.7%, 85.7%, and 100%, respectively. In one case with hypoplastic VA, the detection of flow in the VA by ultrasonography differed from detection by CTA. Meanwhile, there were two cases in which VAs entered at C5 transverse foramen rather than at C6 level. However, ultrasonography could detect the blood flow in these VAs.

**Conclusions:**

Ultrasonography had a sensitivity of 100% compared with CTA in assessment of the VA. Ultrasonography can be used as an initial screening test for VA injury in the emergency room.

**Supplementary Information:**

The online version contains supplementary material available at 10.1186/s12891-023-06426-6.

## Background

Traumatic vertebral artery injury (TVAI) should be considered when patients with cervical spine trauma are taken to the emergency room [1, 2]. This will aid in facilitation of early detection and prevention of ischemic stroke. TVAI occurs in 10–13% of cases of cervical spine trauma, potentially resulting in permanent neurological deficits [3]. Moreover, if the vertebral artery (VA) is occluded due to cervical dislocation, a blood clot may be displaced during surgical repair of the dislocation. Evaluation of the VA is therefore thought to be essential, particularly in emergency surgery for traumatic cervical spine injury.

With improvement of computed tomography (CT) technology, CT angiography (CTA) has been the first choice as an initial screening test for vascular imaging in the head and neck [4]. Remarkable improvement of detection of TVAI has been shown [4], and high sensitivity (98%) and specificity (approximately 100%) have been shown compared with DSA [5]. Magnetic resonance angiography (MRA) is less sensitive than either CTA or DSA in detecting vascular injury [6]. Although CTA has high sensitivity, it also has several disadvantages, including exposure to radiation and the inability to use contrast media in patients with chronic renal failure or contrast media allergies. Transporting patients with multiple traumas to another room for the test is also labor-intensive.

Meanwhile, ultrasonography (US) has reported advantages, including rapid mobility, non-invasiveness, and cost-effectiveness [1]; however, it is only used for the initial evaluation of internal organs in trauma patients in the emergency room. This study aimed to compare US and CTA for the evaluation of the VA in patients with cervical spine injury in the emergency room.

## Methods

### Patient selection

The study protocol was approved by the Wakayama Medical University Research Ethics Committee (approval number: 3449). All patient-related procedures in this study were performed in accordance with the ethical standards of the committee and the 1964 Declaration of Helsinki and its later amendments. A total of 32 consecutive patients with cervical spine injury taken to the emergency room in Wakayama Medical University by ambulance or helicopter between August 2020 and March 2022 and evaluated with US. After exclusion of seven patients who did not also undergo CTA, 25 patients were finally included in the study. All patients provided written, informed consent.

### VA assessment by US

**Fig. 1:**
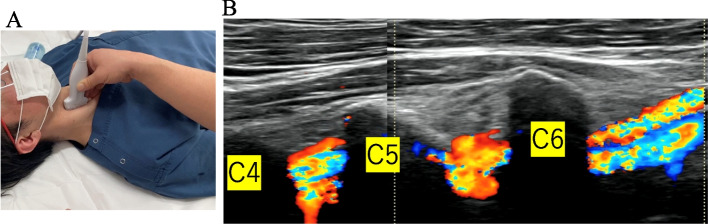
**A** Macro photograph. Ultrasonography examination of a patient in the supine position. **B** Ultrasound image of normal vertebral artery (VA). The VA enters into the C6 bony foramen and passes through C5 and C4 bony foramina

US examination for to assess the VA was performed on the same day the patients were taken to the hospital. Patients were placed in the supine position and facing straight up because of the possibility of cervical instability, and pillows were not placed or removed during the examination. An experienced spine surgeon (Y.I.) searched for VA at levels C4–7 on both sides using color doppler US with a standard ultrasound device (SONIMAGE HS2; Konica Minolta, Tokyo, Japan). A high-frequency linear probe (L11-3; Konica Minolta) provided sufficient visualization (Fig. 1A). In the present study, US was used only to determine the presence of obstruction in VAs, and as soon as blood flow was detected, the US examination was stopped. The ultrasonic probe was placed at the medial border of the sternocleidomastoid muscle. When the longitudinal section of the common carotid artery was shown, the probe was slowly moved laterally until the VA first appeared, and then it was moved down along the VA. After passing through the C6 bony foramen, the VA travels outside the bone at the C7 level. Subsequently, the level of the cervical transverse VA was observed by the examiner, and the sixth to the fourth transverse foramina were identified. The examiner observed whether VA was occluded or not (Fig. 1B). The patient's condition and neck pain were considered during the examination; considering the patient's burden, the examination was terminated as soon as the blood flow was confirmed. We had to keep their heads in the same position, even if it was tilted. We could confirm the C1-3 level from the lateral side of the sternocleidomastoid muscle, but we could not examine the VA on the side in cases in which the heads were tilted, so we examined C4-7 using the described method.

### CTA

After US assessment, CTA was performed for all patients. The same examiner assessed the CTA images irrespective of positive or negative detection of the flow of VA.

### Statistical analysis

All statistical analyses were performed using JMP version 14 software (SAS Inc., Cary, NC, USA). Statistical significance was set at *p* < 0.05. The variability for positivity or negativity of occlusion of the VA or not between US and CTA was confirmed by Kappa analysis.

## Results

We analyzed 50 VAs in 25 patients (19 men and six women, mean age 59.4 ± 16.7). Among these 50 VAs, six were found to be occluded on CTA, and the agreement between US and CTA was 98% (49/50) with 0.92 Cohen’s Kappa index. The sensitivity, specificity, and positive and negative predictive values were 100% (6/6), 97.7% (43/44), 85.7% (6/7), and 100% (43/43), respectively. CTA evaluation of 25 patients at cervical 5–6 vertebral level demonstrated that the diameter of right-side VAs was larger than those on the left (the mean ratio of diameter in both VAs was 1.10).

### Case 1

**Fig. 2:**
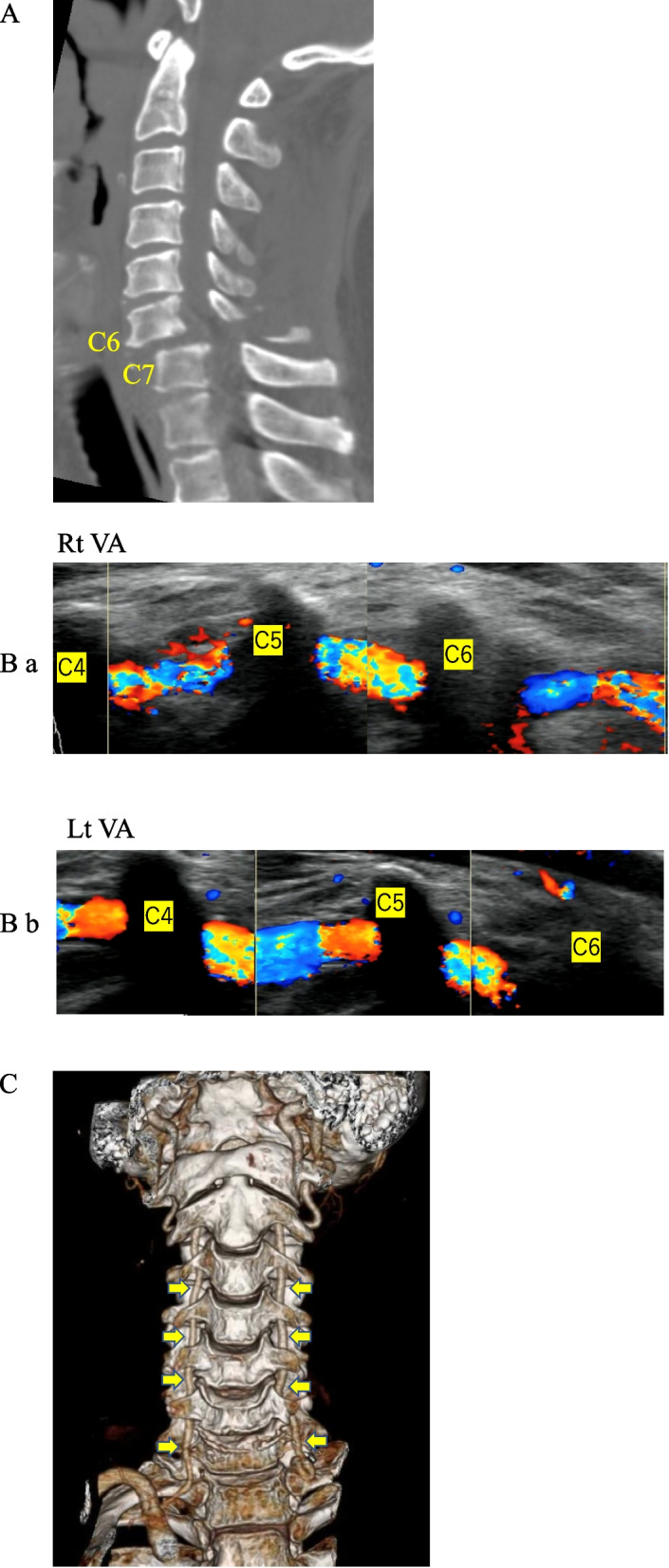
**A** CT shows the dislocation fracture at C6/C7. **B** ab. Ultrasonography shows blood flow in both VAs. **C** CT angiography shows blood flow in both VAs. (Arrow: VA)

A 72-year-old Japanese man fell from a 2-m height and was transferred to the emergency room via helicopter. CT showed bilateral facet dislocation at C6/C7 (Fig. 2A). On US, however, there was obvious VA flow on both sides (Fig. 2B ab). CTA also showed VA flow on both sides, similar to US (Fig. 2C).

### Case 2

**Fig. 3:**
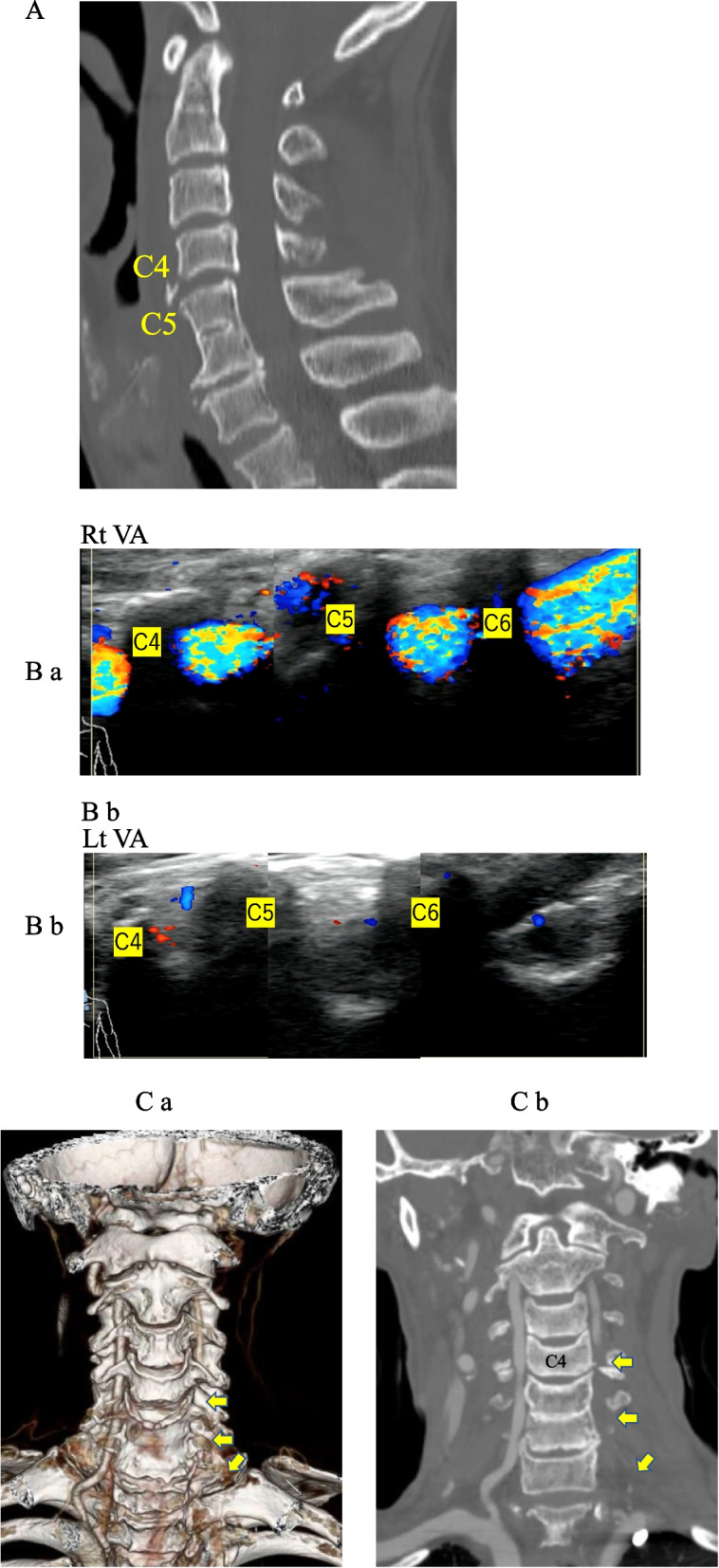
**A** CT shows a teardrop fracture at C4, suggesting hyper-extension injury. **B** ab. US shows no occlusion of the vertebral artery (VA) on the right side, but no flow of VA on the left-side. **C** ab. CTA shows no occlusion of the vertebral artery (VA) on the right side, but no flow of VA on the left side. CTA shows occlusion and flow of VA on the left side but not below C4 level (arrows), but this was not seen by US

A 70-year-old Japanese man fell to the ground while working in the field and was taken to the emergency room by an ambulance. CT showed a teardrop fracture in the C4 vertebral body (Fig. 3A) and MRI also showed a high intensity area in the cervical cord at C4/5 level. The patient had complete paralysis below the C6 level. US showed VA flow on the right side but no flow on the left side (Fig. 3B ab). CTA also showed VA flow on the right side, but there was an occlusion below C4 level on the left side (Fig. 3C ab).

### Case 3

**Fig. 4:**
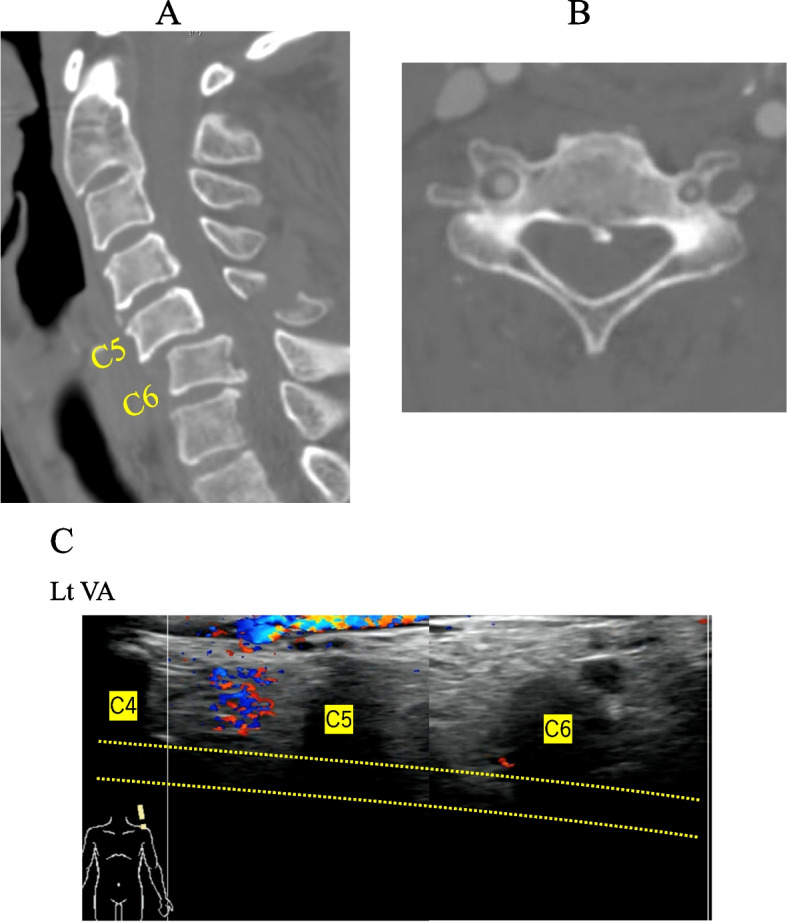
**A** Computed tomography shows the dislocation fracture at C5/C6. **B** CTA shows the stenosis of the left VA at C5 level. The diameter of VA on the left side was 45.9% compared with the one on the right side. **C** Flow of the VA in the left side not seen on US. Part of VA that originally passed through is indicated by dotted lines

There was a case with cervical dislocation (Fig. 4A), in which the diagnosis differed between US and CTA. The left VA was narrower than the right VA at C5 level on CTA, but CTA showed the blood flow in both VAs (Fig. 4B). Meanwhile, the examiner assessed it to be occlusion on the left side by US (Fig. 4C).

### Case 4

**Fig. 5:**
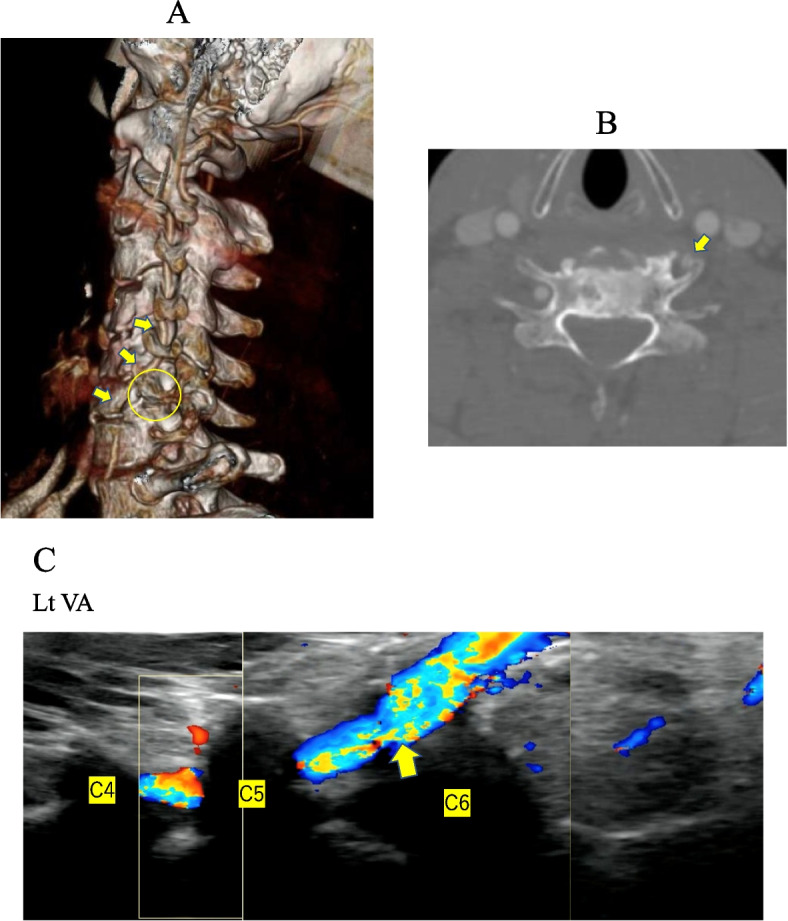
**A** Flow of the right vertebral artery (VA) (arrows). Transverse of the left C6 (circle), with the VA running anterior to it. **B** C6 level. Left vertebral artery (VA) is not transverse to the foramen but anterior (arrow). **C** Left vertebral artery (VA) running anterior to the bony foramen of C6 (arrow)

There were two anomalous cases in which VAs ran anterior to the C6 vertebral body without passing through the C6 foramen before entering at C5 foramen (Fig. 5A and B). We could detect left vertebral artery (VA) running anterior to the bony foramen of C6 (Fig. 5C).

## Discussion

Assessment of the VA is mandatory in patients with cervical spine trauma in order to prevent further cerebral stroke. CTA has been widely used for the evaluation of the VA, but it has various disadvantages that US could compensate for. We therefore tried to evaluate VAs by US in the emergency room and to determine the degree of reliability of US. The results of this study suggest that the agreement between US and CTA was high, particularly all the cases which VA blood flow was confirmed could be done in CTA.

There have been several papers on the assessment of the VA using US, but most of centered upon performance on patients with dizziness, limb disturbance, dysphagia, hemianopia, or other clinical symptoms due to vertebrobasilar insufficiency [2, 7, 8], and none have been centered on the emergency room setting. The accuracy of US in the assessment of the VA has improved over the years, and the sensitivity of CTA has been increasing [8, 9]. Yin et al. reported that as a non-invasive examination, neck-brain integrated US is valuable in the diagnosis of stenotic lesions in cervical VA [8]. However, they also noted that in the diagnosis of VA stenosis, which was divided into four grades (none, mild, severe, and occlusion) there was no significant difference between severe and occlusion in sensitivity, but there was a significant difference between ‘none’ and ‘mild’ in the assessment with CTA.

In this study, there was one case in which the diagnosis was different between US and CTA. CTA may be superior to US when it comes to detecting slight blood flow due to hypoplasia or stenosis by our method. In previous reports on US for examination of VA, the patients had chronic disease and the examiners were free to move the patient’s head position during the examination and to perform several examinations [8, 9]. The accuracy of US for VA in this study was therefore likely to be lower than that for the patients with degenerative disease, in which the head position could not be moved and we performed only one examination. In addition, we cannot detect the diseases like bow hunter’ syndrome [10], which is due to vertebrobasilar insufficiency caused by rotational compression of the VA.

In this study, there were two VAs entering transverse foramen of C5 vertebral body rather than at C6 level. The most common level of entry for the VA into the transverse foramen is at C6, occurring in the majority of patients. Although rare, anatomical variations in the VA entry level have been reported. Bruneu et al., for example, reported VA entries at the C3, C4, C5, or C7 levels, representing 0.2%, 1.0%, 5.0%, and 0.8% of all cases, respectively [11]. In our two cases with VA entry at C5, the blood flow could be confirmed on US.

This study has several limitations. First, US was performed at levels C4–7 only. If there was an occlusion outside of this level, it would not be observed. Second, cases with severe stenosis might be judged to have an occlusion despite the presence of blood flow because of the lack of quantification. Third, the sample size of this study was too small for generalization of the results. Nevertheless, this was the first study to evaluate the agreement between US and CTA in the assessment of TVAI in the emergency room. Moreover, the sensitivity of the tests obtained in this study was sufficient for screening. In future practice, CTA may not be necessary for TVAI at levels C4–7 if VA flow can be confirmed.

## Conclusion

This study showed that US had a sensitivity of 100% compared with CTA in assessment of the VA. Detecting low blood flow due by US may be difficult because of hypoplasia or stenosis of VA. US was useful as an initial screening test for VA injury in the emergency room.

## Supplementary Information

**Additional file 1.**

## Data Availability

The datasets generated during and/or analyzed during the current study are available from the corresponding author on reasonable request. This is not to restrict the use of our dataset, but to allow the Wakayama Medical University Research Ethics Committee to understand the actual use of the dataset.

## Abbreviations

CT: Computed tomography
CTA: CT angiography
DSA: Digital subtraction angiography
MRI: Magnetic resonance imaging
TVAI: Traumatic vertebral artery injury
US: Ultrasonography
VA: Vertebral artery
